# Transition from metal-DTH resistance to susceptibility is facilitated by NLRP3 inflammasome signaling induced Th17 reactivity: Implications for orthopedic implants

**DOI:** 10.1371/journal.pone.0210336

**Published:** 2019-01-17

**Authors:** Lauryn Samelko, Marco S. Caicedo, Joshua Jacobs, Nadim James Hallab

**Affiliations:** Department of Orthopedic Surgery, Rush University Medical Center, Chicago, IL, United States of America; University of Oulu, FINLAND

## Abstract

Metal hypersensitivity has been recognized as an adverse biologic reaction that can compromise total joint arthroplasty (TJA) performance. However, the etiology of metal hypersensitivity responses in TJAs remains unclear. Metal implant debris is known to act as a danger signal that drives NLRP3 inflammasome activation. It remains unknown if implant debris induced inflammasome activation regulates T cell lineage in TJA metal hypersensitivity responses. In this study, we show both *in vivo* and *in vitro* that the pathogenesis of metal hypersensitivity responses to implant debris are largely dependent on activation of the inflammasome/caspase-1 pathway and subsequent production of IL-17A/F by CD4+ T cells. Inhibiting either the inflammasome pathway or IL-17A bioactivity in vivo and in vitro (in vivo using NLRP3 and Caspase-1 deficient mice or in vitro using blocking agents such as Capase-1 inhibitor, IL-1Ra and anti-IL-17A), significantly (p<0.05) mitigated metal-DTH paw inflammation as well as lymphocyte cytokine (IFN-γ and IL-17) and proliferation responses in metal-sensitized mice and primary human PBMCs. This study provides mechanistic insight into how in vivo exposure to orthopedic implant debris, and metals in general, elicits NLRP3 inflammasome activation that mediates the generation of IL-17A/F producing CD4+ T cells, leading to metal-delayed type hypersensitivity reactions.

## Introduction

Total joint arthroplasty (TJA) is a highly successful orthopedic procedure. However, approximately as many as 10–20% of TJAs fail due to well-documented mechanical and biological factors [1–4]. Adverse reactions to metal debris (ARMD) have been identified as a prominent cause of implant failure resulting in revision surgery in metal-on-metal (MoM) hip arthroplasty patients [5–8]. ARMD includes a wide range of periprosthetic soft-tissue reactions such as local soft tissue growths, fibrous pseudotumors, metallosis and toxicity responses. In contrast, another type of response to metal implant debris, histologically identified as aseptic lymphocyte-dominated vasculitis-associated lesions (ALVAL) is identified in periprosthetic tissue as a perivascular lymphocytic infiltration and accumulation of macrophages [9]. ALVAL is also consistent with the diagnosis of cell-mediated type-IV delayed type hypersensitivity (DTH) response [9–16]. Further, patients with high levels of local metal release from failed metal-on-metal total hip replacements (MOM-THR) have been reported as exhibiting increased levels of in vitro metal reactivity with concomitant lymphocyte dominated peri-prosthetic inflammation [14]. Continuing evidence demonstrates a correlation between metal exposure, metal hypersensitivity and implant performance [11, 17–28]. The pathway specific contributions of macrophages and lymphocytes to metal hypersensitivity responses to TJAs remains unclear, despite increasing evidence documenting implant associated metal DTH responses [29–32].

Orthopedic implants are commonly composed of metals such as nickel, cobalt, and chromium. All implants in contact with biological systems generate degradation products (i.e. particulate and soluble metal ions) by wear and corrosion mechanisms [10, 33–39]. Nickel is the most common sensitizer followed by cobalt and chromium, and are commonly associated with metal hypersensitivity responses to metal implants [10, 34–39]. Previous in vivo experimental models of allergic contact dermatitis (ACD) to nickel have shown that epicutaneous exposure to nickel in mice, involves danger signaling via the NLRP3 inflammasome complex but was independent of Toll-like receptor 4 (TLR4) [40]. However, in contrast to metal-ACD models, metal hypersensitivity reactions to TJAs do not involve dermal dendritic cells (DDCs) and Langerhans cells (LC) [41]. Moreover, is not known how models of metal-ACD induced inflammasome activation triggers T-cell subset specific adaptive immune responses, particularly in the case of metal implant debris.

Metal-induced DTH reactions to implant metal exposure have been characterized as generally as CD4+ Th1-cell and IFN-γ mediated with a component of some B-cell participation in a few people [42, 43]. However, this was not always the case since it has been reported that Th2 reactivity to implant Cobalt-alloy (CoCrMo) is also possible, either as a competing or compensatory response [44, 45]. Additional reports have shown that both IFN-γ and IL-17 mRNA expression is exhibited by in vitro stimulated peripheral blood mononuclear cells (PBMCs) in patients with an orthopedic implant that are also reactive to Nickel [46]. This increases the need for determining if mRNA cytokine expression in fact translates to cytokine protein secretion in metal-DTH responses to implant debris. Two central CD4+ Th subsets that play a central role in adaptive autoimmune disease are Th1 cells that secrete IFN-γ and Th17 cells that secrete IL-17A, IL-17F, and IL-17A/F as their signature cytokines [47]. The major determinant of Th cell differentiation is mediated by the presence of cytokine(s) at the point of naïve T cell activation. Th1 cell differentiation is induced by the presence of IL-12 and IFN-γ. While TGF-β, IL-6 or IL-21 selectively induce Th17 cells. Also, IL-1β is a critical signal for the induction and differentiation of CD4+ Th17 cell population in vivo [48]. It is unknown how the initial central mechanism of implant debris reactivity through macrophage (APC) inflammasome activation translates to T-cell subset reactivity, if at all.

Are innate immune pro-inflammatory reactivity (inflammasome reactivity) determinant of specific metal hypersensitivity responses in people with implants Th17 cell mediated? We hypothesized that implant debris induced inflammasome activation promotes IL-17 CD4+ T cell dominant responses in metal hypersensitivity. We report here that implant debris inflammasome activation is a critical step for the induction of IL-17A/F production by CD4+ T cells that elicit metal hypersensitivity responses in TJAs. In inflammasome/caspase-1 deficient metal-sensitized treated mice, there was a significant decrease in IL-17A/F production and effector T cell activity which correspond to a decrease in the severity of metal hypersensitivity responses in vivo, despite the high levels of IFN-γ. In addition, we found that neutralization of the inflammasome pathway or IL-17 in vivo and in vitro, strikingly attenuated the severity of metal-DTH immune reactivity. The pivotal role of both the inflammasome and IL-17 bioactivity in metal-DTH responses was further supported using in vitro testing of metal-reactive human primary lymphocytes. These data demonstrate that implant debris induced inflammasome activation tips the balance toward inflammatory IL-17A/F producing CD4+ T cells that drive metal hypersensitivity responses in TJAs.

## Materials and methods

### Ethics statement

This study and consent process received approval from the Rush University Institutional Review Board.

### Animal research

Female 10–12 week old C57BL/6 and Caspase-1-/- (C57BL/6 background) mice were obtained from The Jackson Laboratory (Bar Harbor, Maine) and NLRP3-/- (C57BL/6 background) were obtained from University of Lausanne. Mice were provided a standard laboratory diet and water, and maintained under pathogen-free conditions under a 12-hour light/dark cycle. All experiments were carried out under the guidelines of the Institutional Animal Care and Use committee at Rush University Medical Center. The protocol was approved by the committee on the Ethics of Animal Experiments of Rush University Medical Center (IACUC) and all experiments in our study were conducted adhering to the institution’s guidelines for animal husbandry, and followed the guidelines and basic principals in the Public Health Service Policy on Humane Care and Use of Laboratory Animals, and the Guide for the Care and Use of Laboratory Animals, United States Institute of Laboratory Animal Resources, National Research Council. All efforts were made to minimize suffering; all manipulations were performed under isoflurane and mice were sacrificed by cervical dislocation after being administrated isoflurane.

### Media and reagents

Isolated murine and human cells were cultured with sterile RPMI 1640 supplemented with L-Glutamine, Penicillin, Streptomycin, 25mM Hepes (Lonza, Walkersville, MD).

Nickel (II) chloride (NiCl_2_), cobalt (II) chloride (CoCl_2_), phorbol 12-myristate 13-acetate (PMA), ionomycin, phytohemagglutinin (PHA), and Mitomycin-C were purchased from Sigma Aldrich (St. Louis, MO). NiCl_2_ and CoCl_2_ were reconstituted in sterile water and stock solutions were freshly prepared for each in vitro and in vivo experiment. Complete freund’s adjuvant (CFA) was purchased from InvivoGen (San Diego, CA).

### In vivo delayed type hypersensitivity (DTH) to orthopedic implant metal(s)

DTH responses are comprised of two phases: 1) sensitization and 2) effector phase. (1) Sensitization phase to implant metal(s): To induce DTH with orthopedic implant metal(s), mice were injected with 125μl intraperitoneally (i.p) on day 1 and day 10, with either an emulsion of the adjuvant CFA with sterile PBS at equivolume (control group) or an emulsion of CFA with soluble 10mM stock concentration of NiCl_2_ and CoCl_2_ (metal-sensitized group) for initial metal sensitization (previously shown that NiCl_2_ is most effective sensitizer at this concentration)[49, 50]. I.P. injections did not cause any keloid lesions at the injection site. (2) Effector phase to implant metal(s): Re-exposure to the metal allergen leads to DTH reaction during the effector phase. Therefore, on day 12, 50μl mixture of either sterile PBS (control group) or NiCl_2_ (metal-sensitized group) with an equivolume of CFA was injected subcutaneously on top of the right paw. DTH was determined 48 h post re-exposure to metal allergen (day 14) by measuring changes in paw inflammation using a digital caliber (blinded assessment) and the induced difference was recorded. Each group consisted of 3–5 mice, experiments were performed at least three independent times.

### Histology

Murine paws were fixed with 10% phosphate buffered formaldehyde. Paraffin-embedded tissue anterior-posterior (AP) plane 5um sections were stained with H&E using standard techniques.

### Lymphocyte transformation test (LTT) assay for determination of *in vitro* metal reactivity

In addition to determination of paw swelling after recall with NiCl_2_, proliferation in vitro of lymphocytes was measured. Spleens were obtained aseptically 48 h after challenge and splenic effector CD4+ T cells were isolated using negative selection kit following manufacturer’s protocol (EasySep Mouse CD4+ T cell isolation kit; 2.5X10^5^ cells/per well in 96 flat bottom plate). Splenic CD4+ T cells were co-incubated with syngeneic mitomycin-C treated naïve splenic (2.5X10^5^ cells/per well) antigen-presenting cells (APCs) with or without 0.0001mM or 0.001mM of soluble metal ions NiCl_2_ or CoCl_2_, and 0.01 mg/ml phytohemagglutinin (PHA) as a positive control, in RPMI 1640 media containing 10% fetal bovine serum (FBS; Hyclone Laboratories, Inc.) in 37°C and 0.5% CO_2_ for 4 days. Syngeneic naïve spleen APCs were incubated at 5X10^6^ cells/mL with 25 μg/mL of mitomycin-C for 30 minutes to inactivate proliferation. At day 3 of cell culture, ^3^H-thymidine was added. At day 4, T-cell proliferation was assessed via ^3^H-thymidine incorporation was determined using a beta scintillation counter.

Human lymphocyte isolation: De-identified human whole blood, not taken for the specific purpose of this study, was obtained via venipuncture, under study specific IRB approval. De-identified human peripheral blood mononuclear cells were isolated from 30 mL of peripheral blood using density gradient separation (Ficoll-isopaque, Pharmacia, Piscataway, NJ). Ficoll gradient separated mononuclear cells are generally comprised of 85–95% lymphocytes with 5–13% monocytes and <0.1% dendritic cells with limited contamination (i.e. <5% erythrocytes and <3% granulocytes). Collected PBMCs (white buffy coat) were washed in sterile phosphate-buffered saline (PBS) solution and resuspended in RPMI-1640 medium with 10% autologous serum and cultured with either no metal (plain media) as a negative control, PHA as a positive control, or with soluble nickel (NiCl_2_) or cobalt (CoCl_2_) in 37°C and 0.5% CO_2_ for 6 days. At day 5 of cell culture, ^3^H-thymidine was added. At day 6, T-cell proliferation was assessed via ^3^H-thymidine incorporation was determined using a beta scintillation counter.

### Anti-mouse-IL-1R local treatment

Anti-mouse IL-1R: A set of metal-sensitized C57BL/6 female mice received local paw administration on day 12 with 200 μg of anti-mouse IL-1R1 (for local in vivo blocking of IL-1R during the effector phase of metal-DTH responses) [51, 52] (clone JAMA-147;BioLegend, San Diego, CA) in concert with NiCl_2_ and CFA.

### Anti-mouse-CD86 treatment

Anti-mouse CD86 MAb treatment: Mice were treated with 400 μg of InVivoMAb anti-mouse CD86 (B7-2; BioXCell, West Lebanon, NH) in 200 μl PBS starting at day 1 of metal sensitization and subsequently received 200 μg of anti-mouse CD86 in PBS after 48 hours and every 48 hours until the experiment was terminated on day 14 [53, 54].

### Anti-mouse-IL-17A treatment

Anti-mouse IL-17A MAb treatment: Mice were treated with 400 μg of InVivoMAb anti-mouse IL-17A (BioXCell, West Lebanon, NH) in 200 μl PBS starting at day 1 of metal sensitization and subsequently received 200 μg of anti-mouse IL-17A in PBS after 48 hours and every 48 hours until the experiment was terminated on day 14 [55].

### In vitro treatment of human lymphocytes with inflammasome and IL-17A inhibitor(s)

Inflammasome inhibitor(s): Isolated human PBMCs were co-incubated in vitro with either MCC950, a NLRP3 inflammasome inhibitor (InvivoGen), Caspase-1 inhibitor (Caspase-1/ICE inhibitor Z-WEHD-FMK) or recombinant human IL-1Ra (R&D Systems. Inc., Minneapolis, MN) for metal-LTT assay or cytokine analysis according to manufacturer’s protocol and working concentration [56].

### IL-17A Monoclonal Antibody

Isolated human PBMCs were co-incubated in vitro with IL-17A monoclonal antibody (eBio64CAP17; ThermoFisher Scientific) for neutralization of IL-17A bioactivity for metal-LTT assay or cytokine analysis according to manufacturer’s protocol and working concentration.

### Cytokine analysis

Sandwich ELISAs for mouse IL-17A/F and IFN-γ (R&D Systems. Inc., Minneapolis, MN) were used to detect cytokine production in both non-sensitized and metal-sensitized female mouse spleens and isolated CD4^+^ T-cells, that were harvested 48h post paw re-challenge to metal allergen (d 14). Supernatants from control and metal-challenged murine cells were collected after 4 d. Sandwich ELISAs for human IL-17A/F and IFN- γ (R&D Systems. Inc., Minneapolis, MN) were used to detect cytokine production in supernatants from isolated control and metal-challenged human PBMCs that were collected after 5 d. All samples for ELISA were performed in triplicate in 96 well plates and were stored at -80°C until analysis, following manufacturer’s protocol.

### Statistical analysis

D’Agostino and Pearson omnibus normality test was performed to determine normality for each of the data sets. If the data set passed the normality test (alpha = 0.05), then data was subsequently analyzed using Fisher’s LSD for comparison of groups post-hoc after significance was determined with ANOVA, and is represented as the mean (+SEM) from triplicate data from three independent experiments. To determine statistical significant differences among intragroup comparisons, two-tailed paired Student’s t-test was used and for intergroup comparisons, unpaired Student’s t-test was used as appropriate. Mann-Whitney was used for the comparison of two groups and Kruskal-Wallis for the comparison of more than two data sets for non-normal distributed data and/or n<15 data points[57]. All statistical analysis was performed using Prism 6.0 program (GraphPad, San Diego, CA). Statistical difference was considered significant at p ≤ 0.05.

## Results

### *In vivo* induction of DTH responses to orthopedic metal implant debris

**Fig 1:**
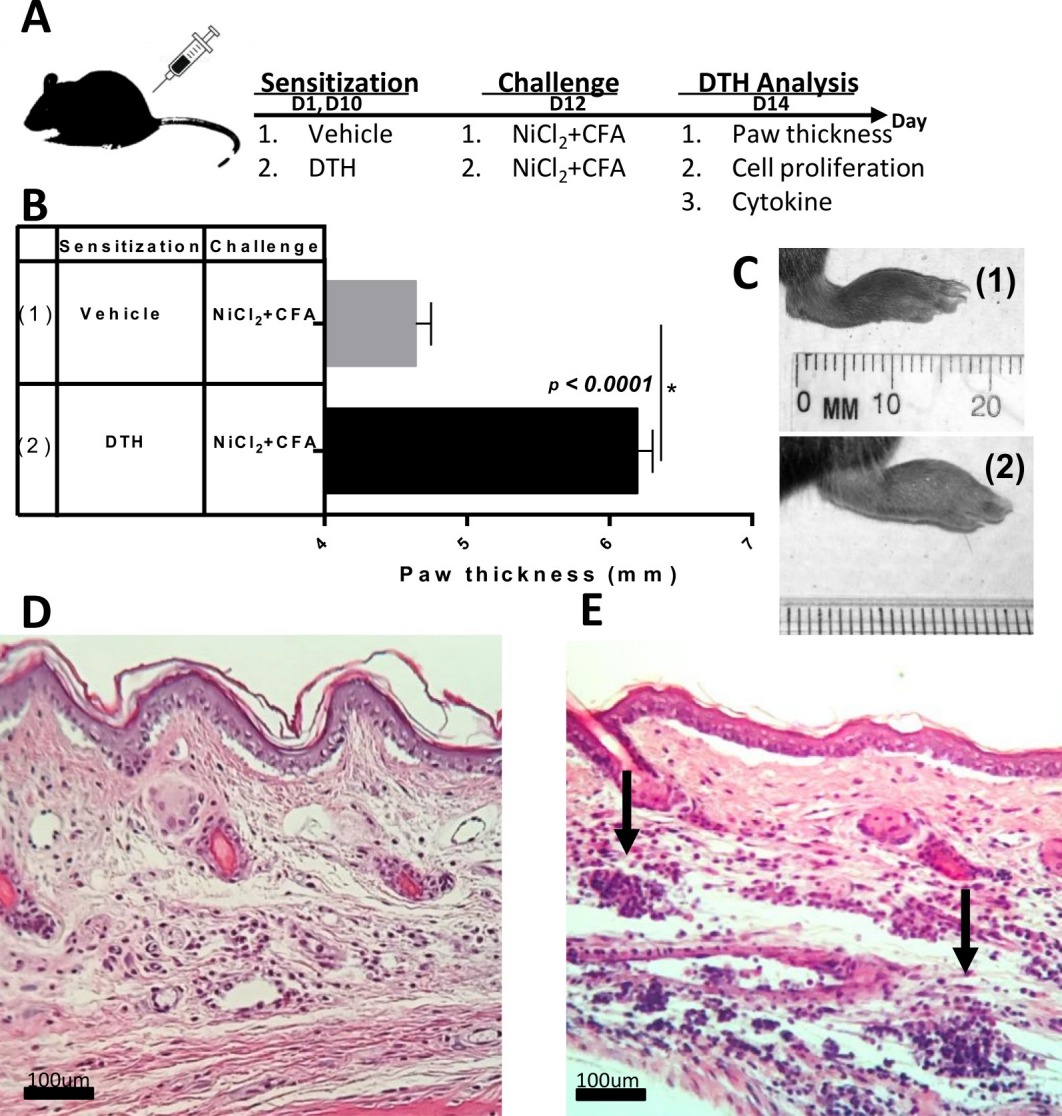
*In vivo* exposure to orthopedic implant metal(s) induces metal-DTH responses. (A) Schematic model for generating metal-DTH to orthopedic implant metal(s) in vivo. (B) DTH was determined by measuring paw thickness 48 h after challenge (D 14) in C57BL/6 mice that were sensitized and challenged as indicated on D 12 and (C) corresponding representative photographs of inflammatory lesions in the paw of vehicle and metal-DTH mice on D 14. C57BL/6 mice that were paw challenged without prior sensitization, vehicle group (Group 1), served as negative controls. Representative paw histology sections from (D) vehicle-treated or (E) DTH-treated C57BL/6 mice, demonstrating inflammatory lymphocytic infiltrations (arrows) found in the paw tissue of DTH mice. Data represent three independent experiments with four mice/group in each experiment. The amount of cell proliferation is represented as the mean of counts per minute (cpm) ± SEM. Statistical significance was determined by Student’s unpaired two-tailed t-test and asterisk (*) denote significant differences P≤0.05.

Recent prospective studies have shown that the rate and degree of metal sensitization in patients with MoM hip arthroplasties is increased compared to the general population, supporting the contention that metal-induced lymphocyte reactivity increases with increased metal exposure [28]. To determine if in vivo exposure to clinically relevant implant metal degradation productions (i.e. metal ions) induces metal-DTH responses, we first sensitized female C57BL/6 mice with an intraperitoneal (i.p.) injection of a mixture of CFA, NiCl_2_ and CoCl_2_ at day 1 and day 10 (**Fig 1A**) [50]. Subsequent re-exposure/challenge to metal allergen (NiCl_2_) leads to a DTH reaction during the effector phase. To assess if mice developed a DTH reaction to orthopedic metals, mice were challenged on day 12 by a subcutaneous injection onto their paw superficially to NiCl_2_ with CFA. Mice in the vehicle group that received paw challenge without prior metal sensitization served as negative controls. On day 14, metal-DTH responses were evaluated and measured by a blind assessment of localized paw inflammation via a digital caliber (**Fig 1B**). Upon challenge to Ni, sensitized-mice revealed a greater severity of a metal-DTH response as measured by significant increase in paw inflammation (6.195 mm mean paw inflammation; p<0.0001) as compared to vehicle group. Additionally, redness and swelling were only observed in the paw(s) of metal-sensitized C57BL/6 mice (**Fig 1B and 1C**). Histological examination revealed dramatic differences in the infiltration of lymphocytes in the paw tissues of sensitized mice (**Fig 1D vs. 1E**). These results indicate that in vivo exposure to implant metal degradation products can lead to robust metal-DTH responses.

**Fig 2:**
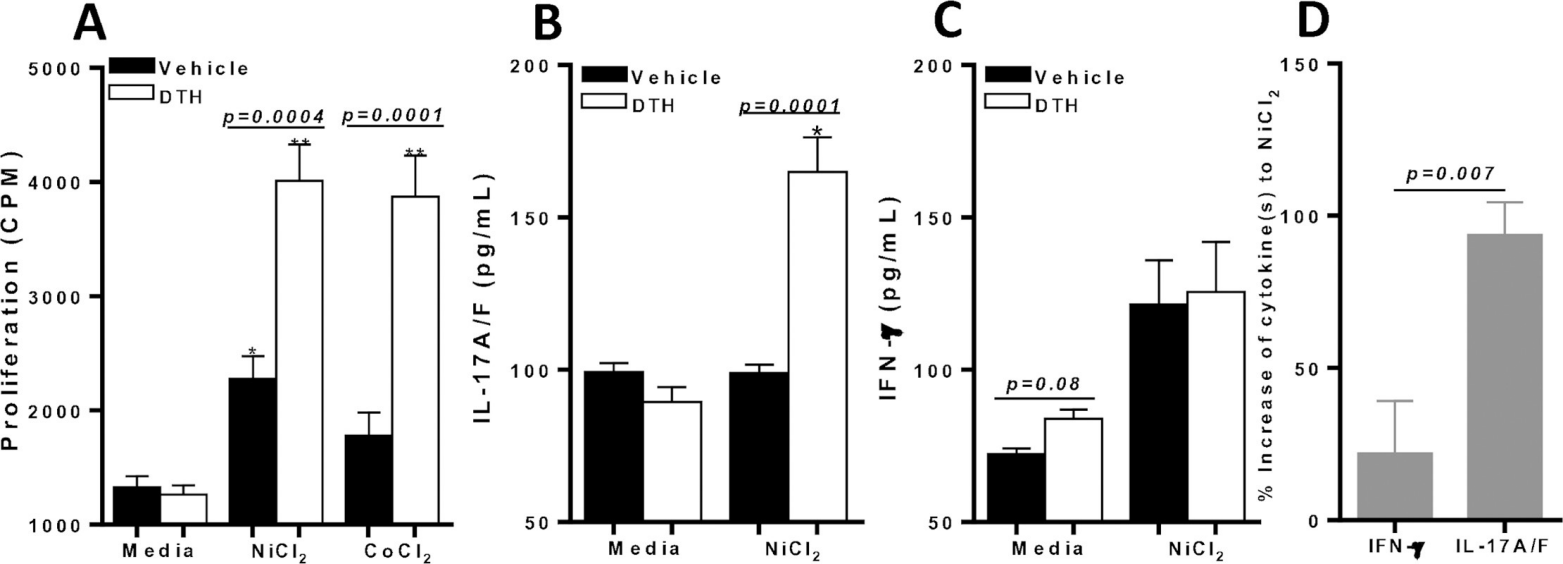
Metal-DTH responses to implant debris in metal-sensitized C57BL/6 mice induces metal-reactive CD4+ T cells. At D 14, spleens were harvested. CD4+ T cells were purified from mouse spleens and co-cultured with mitomycin-C treated naïve total spleen cells with or without Ni (0.001mM) or Co (0.001mM) challenge for four days. (A) Proliferation of CD4+ T cells was measured by ^3^H-thymidine incorporation. Supernatants were harvested and assayed by ELISA for (B) IL-17A/F and (C) IFN-γ. (D) Percentage increase of produced cytokines in response to Ni challenge, calculated using cytokine production from Fig 1D and 1E from DTH vs. vehicle treated mice. Data represent one of three independent experiments with four mice/group in each experiment. Data are shown as mean ± SEM. Statistical significance was determined by Student’s unpaired two-tailed t-test and asterisk (*) denote significant differences P≤0.05.

In addition to determining paw swelling after recall with NiCl_2_, proliferation in vitro of splenic CD4+ T cells from vehicle and DTH mice was measured. CD4+ T cells from metal-DTH mice displayed a significant increase in NiCl_2_ and CoCl_2_ specific proliferation than CD4+ T cells from vehicle-treated mice (**Fig 2A**). The proliferative response of CD4+ T cells from metal-DTH treated mice corresponded with significant increase in IL-17A/F production to Ni (**Fig 2B**). However, IFN-γ secretion by CD4+ T cells from metal-DTH treated mice was non-significantly increased to Ni (**Fig 2C**). In contrast, CD4+ T cells from vehicle-treated mice did not exhibit any increase in IL-17A/F production but exhibited a non-significant increase in IFN-γ production to Ni (p = 0.07; Fig 2B and 2C). These data demonstrate that metal-sensitized mice have a CD4+ T cell specificity recall response to Ni that is IL-17A/F mediated.

### Inflammasome activation is required to elicit a metal-DTH response in sensitized mice

**Fig 3:**
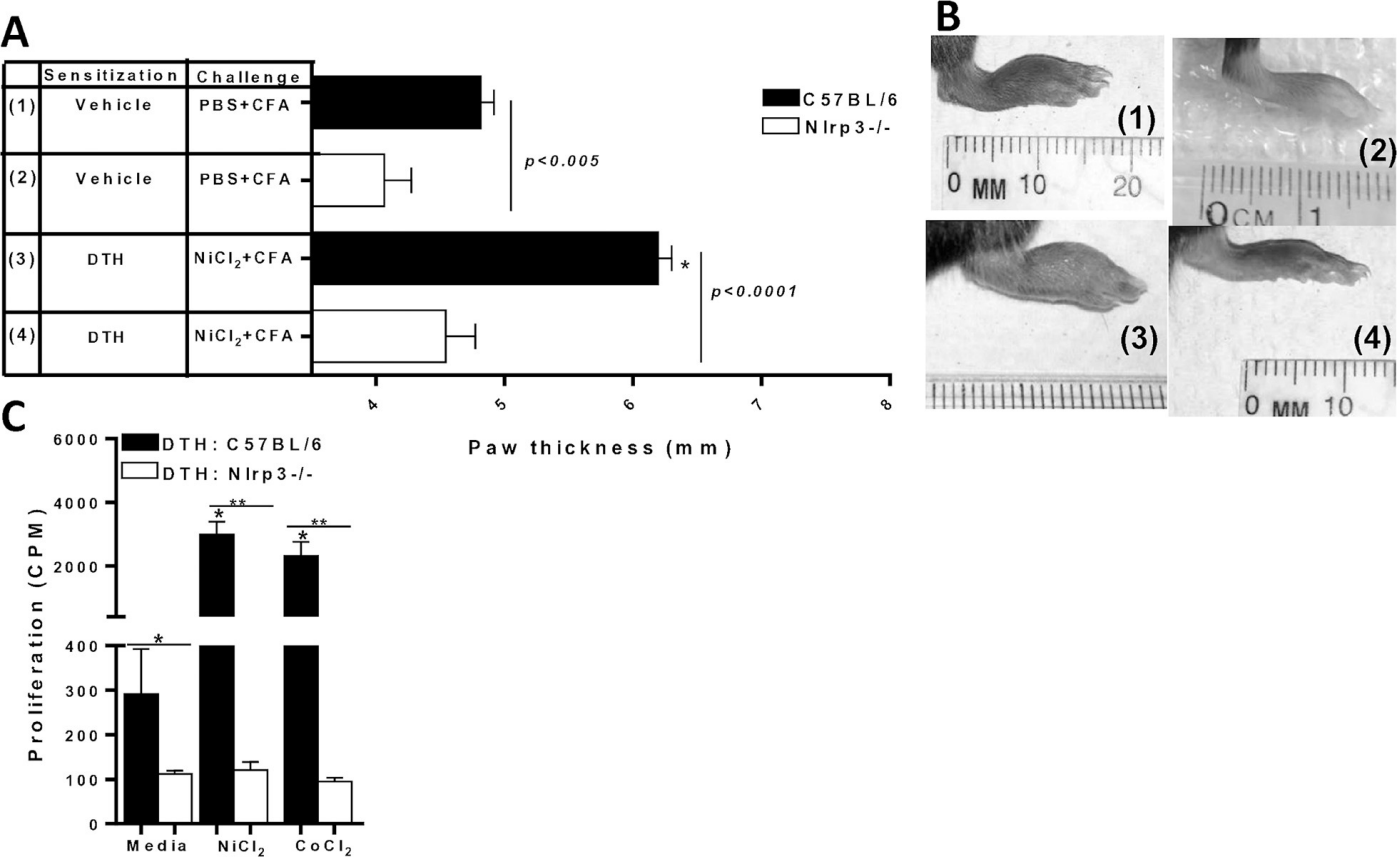
Nlrp3-/- mice do not exhibit metal-DTH reactivity *in vivo* or *in vitro*. (A) DTH was determined by measuring paw thickness 48 h after challenge (D 14) in mice that were sensitized and challenged as indicated on D 12 and (B) corresponding representative photographs (correspond to label group numbers 1–4) of inflammatory lesions in the paw of vehicle- and metal-DTH treated C57BL/6 (black bars) or Nlrp3-/- (white bars) mice. At D 14, spleens were harvested co-cultured with or without Ni (0.001mM) or Co (0.001mM) challenge for four days. (C) Proliferation of splenocytes was measured by ^3^H-thymidine incorporation. Data represent one of three independent experiments with 3–4 mice/group in each experiment. Data are shown as mean ± SEM. Statistical significance was determined by Student’s unpaired two-tailed t-test (* P≤0.05, ** P≤0.001).

To evaluate the effect of NLRP3 inflammasome on metal-DTH responses, Nlrp3-/- mice were sensitized using our model system to induce metal-DTH responses (Fig 1A). The mean paw thickness of both vehicle-treated and metal-sensitized-treated Nlrp3-/- mice was significantly decreased compared with that of vehicle and metal-DTH C57BL/6 mice (**Fig 3A and 3B**). Redness and swelling was not observed in the paw of Nlrp3-/- mice (Fig 3B). Further, proliferation in vitro of isolated splenic cells from metal-DTH Nlrp3-/- mice displayed a significant decrease in NiCl_2_ and CoCl_2_ specific proliferation compared with metal-DTH C57BL/6 mice (**Fig 3C**).

**Fig 4:**
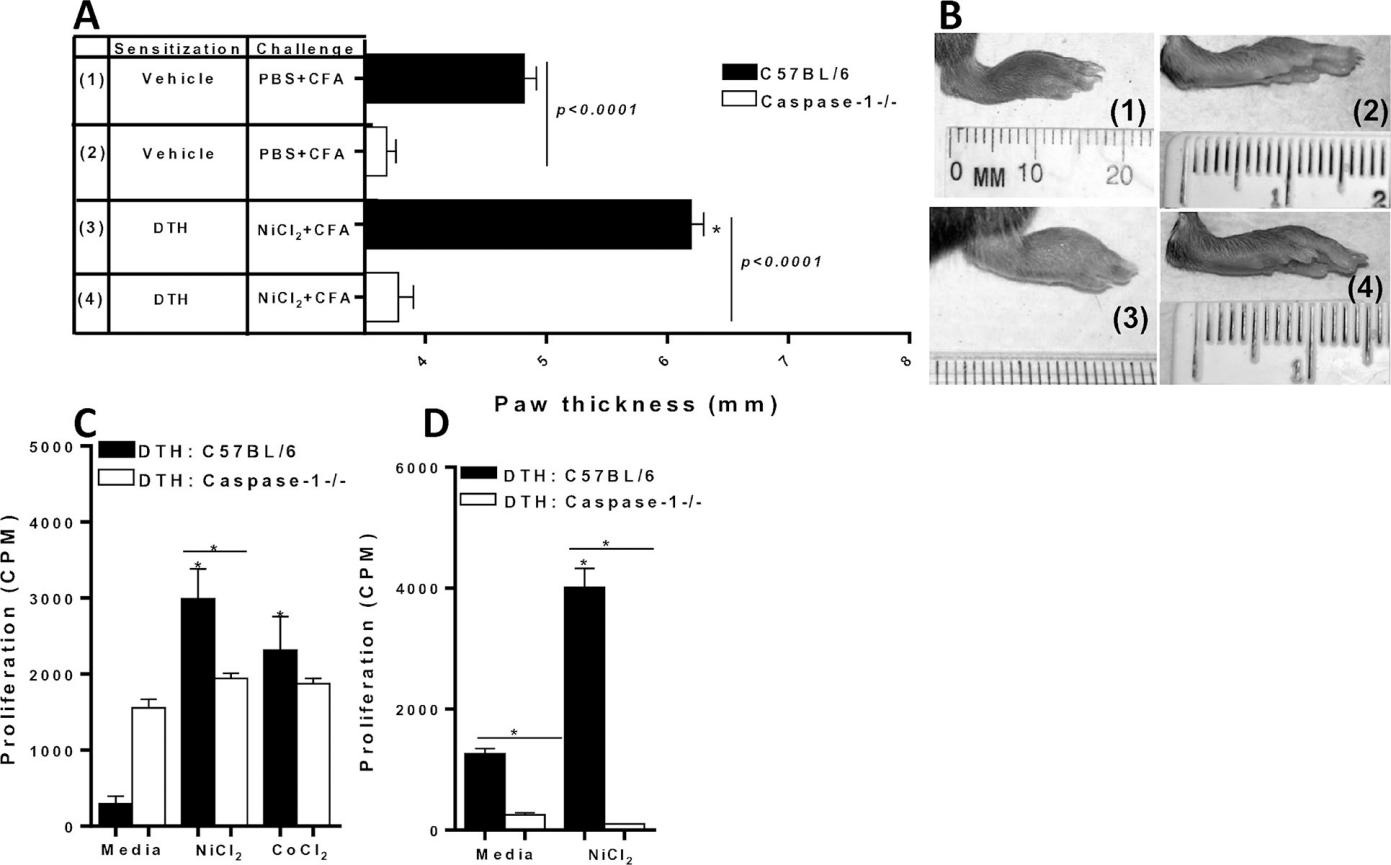
Caspase-1-/- mice do not exhibit metal-DTH reactivity *in vivo* or *in vitro*. (A) DTH was determined by measuring paw thickness 48 h after challenge (D 14) in mice groups 1–4 that were sensitized and challenged as indicated on D 12 and (B) corresponding representative photographs (correspond to label group numbers 1–4) of inflammatory lesions in the paw of vehicle- and metal-DTH treated C57BL/6 or Caspase-1-/- mice. At D 14, spleens were harvested co-cultured with or without Ni (0.001mM) or Co (0.001mM) challenge for four days. (C) Proliferation of splenocytes was measured by 3H-thymidine incorporation. CD4+ T cells were purified from either DTH C57BL/6 or DTH Caspase-1-/- mouse spleens and co-cultured with mitomycin-C treated naïve total spleen cells from C57BL/6 or Caspase-1-/- mice respectively, with Ni (0.001mM) challenge for four days. (D) Proliferation of CD4+ T cells was measured by ^3^H-thymidine incorporation. Data represent one of three independent experiments with 3–4 mice/group in each experiment. Data are shown as mean ± SEM. Statistical significance was determined by Student’s unpaired two-tailed t-test (* P≤0.05).

NLRP3 inflammasome mediates the activation of caspase-1, which promotes the production of pro-inflammatory IL-1beta. Thus, the rate-limiting step in inflammation due to IL-1beta is the activation of caspase-1. To further assess the role of the inflammasome pathway, Caspase-1-/- mice were metal-sensitized. Metal-DTH Caspase-1-/- mice exhibited significantly less paw inflammation compared with C57BL/6 mice (**Fig 4A and 4B**). Further, metal-DTH Caspase-1-/- mice had less paw inflammation (mean paw thickness = 3.77 mm) compared with metal-DTH Nlrp3-/- mice (mean paw thickness = 4.54 mm; Fig 3A vs. 4A). Caspase1-/- metal-DTH mice also displayed significant decreases in both splenic and CD4+ T cell proliferation to metal challenge (**Fig 4C and 4D**). These results reveal that metal-DTH responses to implant debris are dependent on active NLRP3 inflammasome and caspase-1 signaling in vivo and in vitro. However, this data also reveals that immune reactivity during a metal-DTH response, are more dependent on active caspase-1 signaling than the NLRP3 inflammasome.

### Metal–sensitized Caspase-1-/- deficient mice exhibit robust production of IFN-γ to Ni

**Fig 5:**
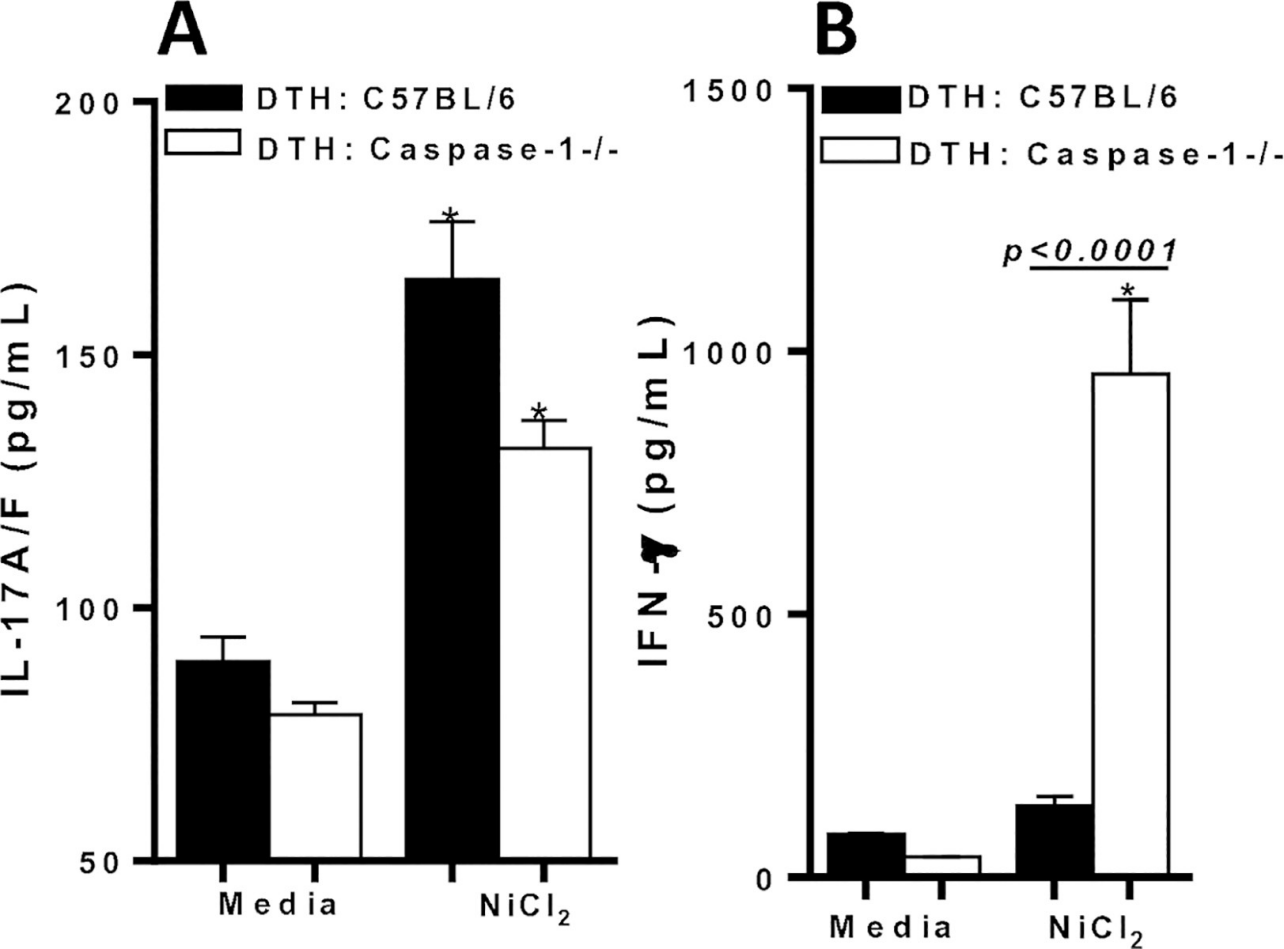
Cytokine production by CD4+ T cells from metal-DTH C57BL/6 and Caspase-1-/- mice. At D 14, spleens were harvested from metal-sensitized (DTH) C57BL/6 (black bars) and Caspase-1-/- (white bars) mice. CD4+ T cells were purified from mouse spleens and co-cultured with mitomycin-C treated naïve total spleen cells from either C57BL/6 or Caspase-1-/- mice respectively, with or without Ni (0.001mM) challenge for four days. Supernatants were harvested and assayed by ELISA for (A) IL-17A/F and (B) IFN-γ. Data represent one of three independent experiments with four mice/group in each experiment. Data are shown as mean ± SEM. Statistical significance was determined by Student’s unpaired two-tailed t-test and asterisk (*) denote significant differences P≤0.05.

CD4+ T cells isolated from metal-sensitized Caspase-1-/- mice exhibited a significant increase of IL-17A/F expression to Ni (mean secretion = 131.5 pg/mL; **Fig 5A**). Also, IFN-γ production by CD4+ T cells from metal-sensitized Caspase-1-/- mice was significantly increased to Ni (mean secretion = 956.6 pg/mL), and compared to metal-sensitized C57BL/6 mice (p<0.0001; **Fig 5B**). However, IFN-γ production was significantly greater than IL-17A/F production in metal-sensitized Caspase-1-/- mice to Ni (p = 0.006; comparing data from Fig 5A to 5B). The IL-17 secretion was likely suppressed due to the lack of inflammasome/caspase-1 signaling, and the ability of IFN-γ to suppress inflammatory IL-17 production. These data indicate that the absence of caspase-1 activity leads to the abatement of metal-DTH responses, likely due to significant increases in IFN-γ production that suppress Ni induced IL-17 production.

### Ni-specific CD4+ T cells require active caspase-1

**Fig 6:**
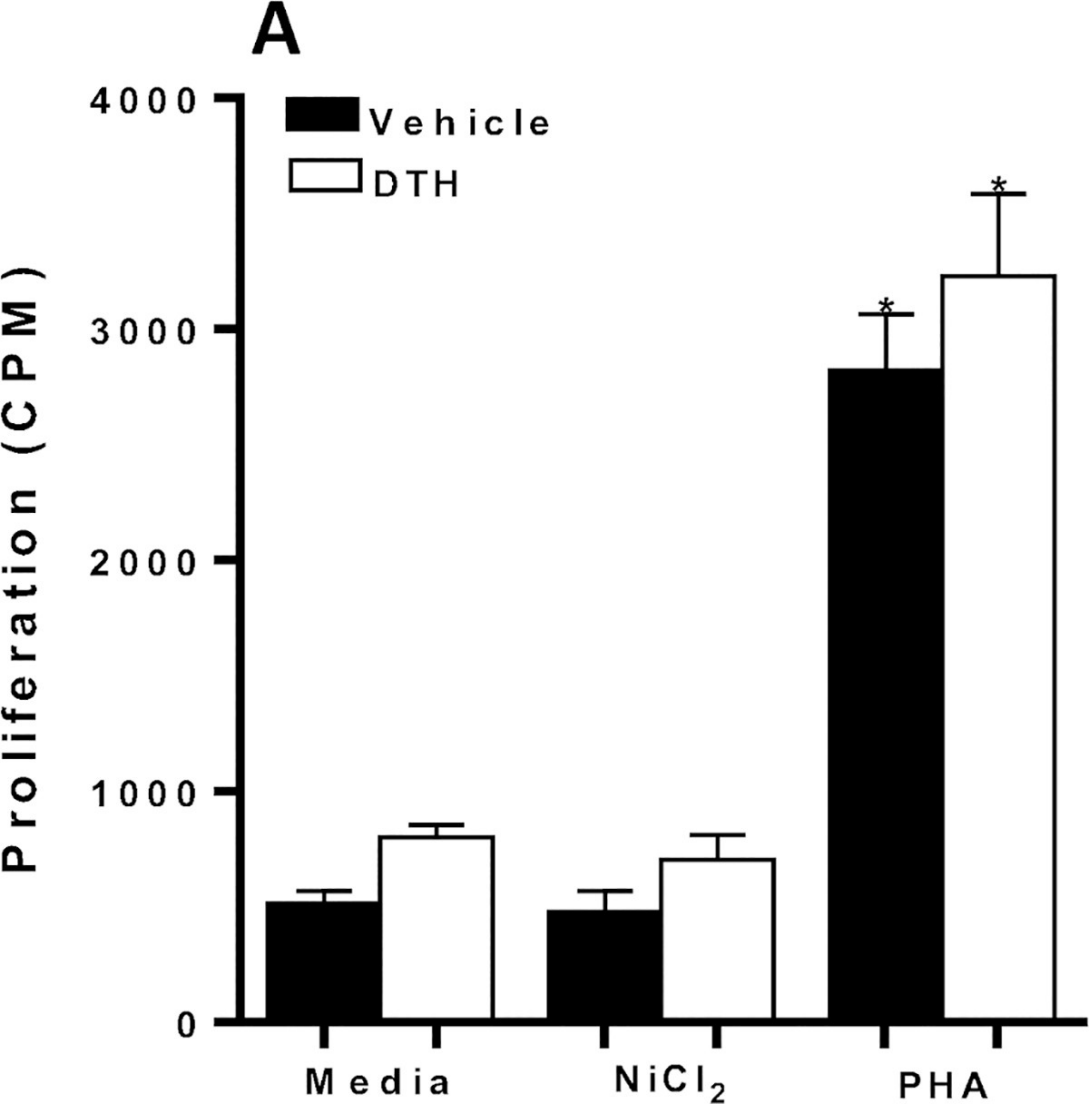
Metal-reactive CD4+ T cells require caspase-1 activity by APCs. At D 14, spleens were harvested from vehicle and metal-sensitized (DTH) C57BL/6 mice. CD4+ T cells were purified from mouse spleens and co-cultured with mitomycin-C treated naïve total spleen cells from Caspase-1-/- mice, with Ni (0.001mM) challenge or PHA (non-specific mitogen stimuli) for four days. (A) Proliferation of CD4+ T cells was measured by ^3^H-thymidine incorporation. Data represent one of three independent experiments with four mice/group in each experiment. Data are shown as mean ± SEM. Statistical significance was determined by Student’s unpaired two-tailed t-test and asterisk (*) denote significant differences P≤0.05.

We next assessed if Ni-specific CD4+ T cell proliferation was dependent on active caspase-1 activity by antigen presenting cells (APCs). CD4+ T cells from metal-sensitized C57BL/6 mice were co-incubated with mitomycin-c treated APCs (mitomycin-C inhibits cell proliferation and allows for the measurement of only the proliferation of the CD4+ T cell population) from naïve Caspase-1-/- mice in the presence or absence of Ni (**Fig 6**). CD4+ T cells from vehicle and metal-sensitized C57BL/6 mice failed to proliferate in response to Ni. However, CD4+ T cells displayed significant proliferative response to PHA, a nonspecific mitogen stimulus. These results suggest that APCs require caspase-1/IL-1 activity to effective prime and activate Ni-specific IL-17 producing CD4+ T cells in metal-DTH.

### Ni-specific CD4+ T cells require IL-1 signaling

**Fig 7:**
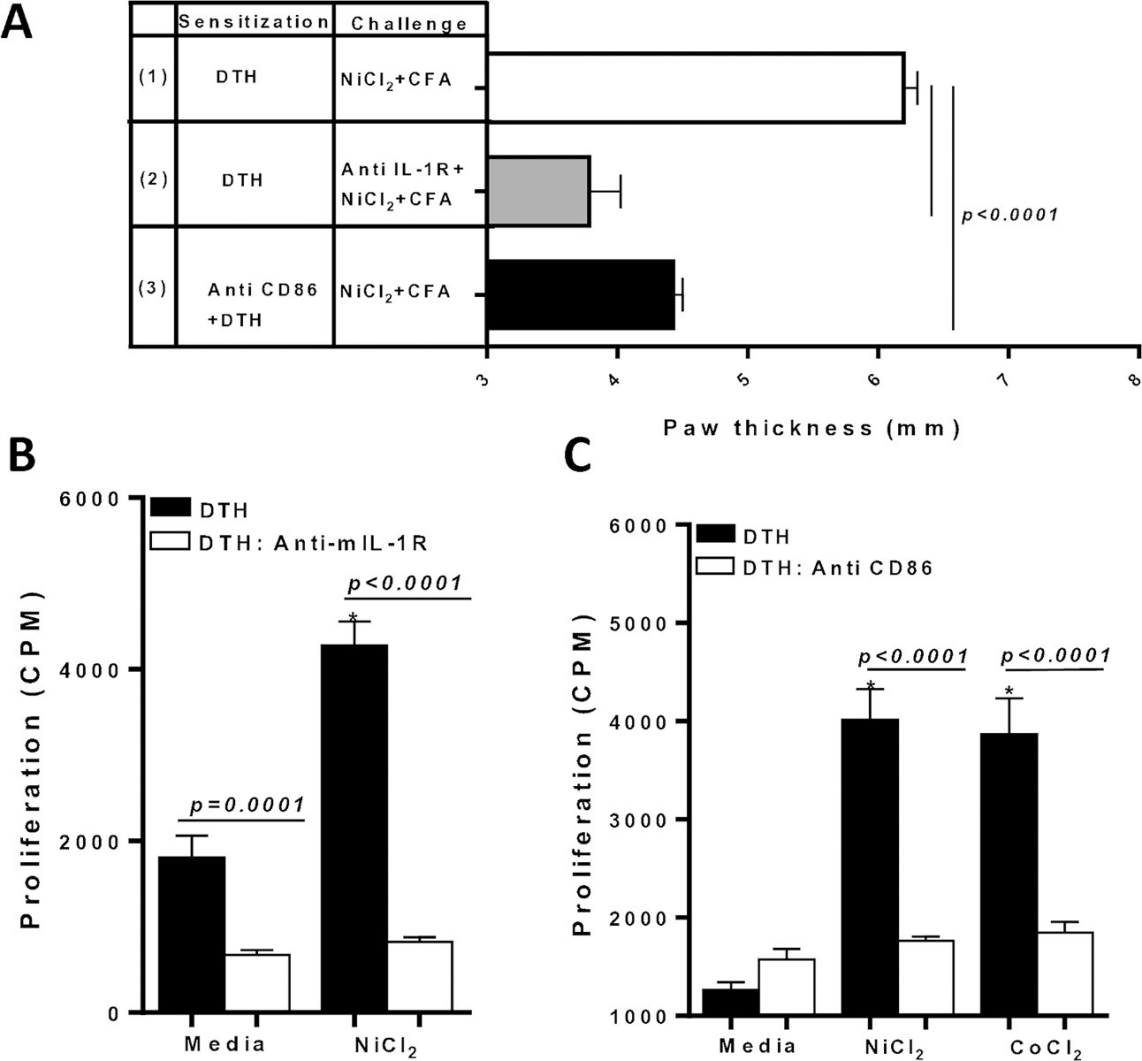
IL-1 and costimulatory signal by APCs are required for *in vivo* metal sensitization and for in vitro metal-reactive CD4+ T cells. (A) DTH was determined by measuring paw thickness 48 h after challenge in C57BL/6 mice that were sensitized and challenged as indicated on D 12. Sensitized mice in group 2 received local administration of antimIL-1R at the same time as challenge was delivered to the paw on D 12. Sensitized mice in group 3 received systemic ip injection of Anti-CD86 prior to metal sensitization and every 48 hrs until D 14. At D 14, spleens were harvested. CD4+ T cells were purified from the spleens of either DTH C57BL/6, (B) DTH C57BL/6 mice treated locally with antimIL-1Rr or (C) DTH C57BL/6 mice treated systemically with Anti-CD86 and co-cultured with mitomycin-C treated naïve total spleen cells from C57BL/6 with Ni (0.001mM) or Co (0.001mM) challenge for four days. Proliferation of CD4+ T cells was measured by ^3^H-thymidine incorporation. Data represent one of three independent experiments with 3–4 mice/group in each experiment. Data are shown as mean ± SEM. Statistical significance was determined by Student’s unpaired two-tailed t-test (* P≤0.05).

Since caspase-1 is the rate limiting step in inflammatory response mediated by IL-1β, and caspase-1 activity is central to our murine model of metal-DTH responses, we next assessed if IL-1R signaling is also necessary to promote metal-DTH responses. Metal-sensitized C57BL/6 received local administration of anti-mIL-1R onto their paw in concert with challenge of Ni and CFA on day 12. Anti-IL-1R treated metal-sensitized mice displayed significantly less paw inflammation compared to metal-sensitized C57BL/6 mice (p<0.0001; **Fig 7A**). Further, CD4+ T cells from anti-IL-1R treated metal-sensitized mice had significantly less proliferation to Ni compared with CD4+ T cells from metal-sensitized mice (p<0.0001; **Fig 7B**). These results demonstrate that Ni-specific CD4+ T cell responses also require IL-1 inflammatory activity.

### Ni-specific CD4+ T cells require CD86 co-stimulation

We next asked whether CD4+ T cells require co-stimulation signals for induction of metal-reactive T cells and onset of metal-DTH responses. To determine if costimulatory molecules have a central role in the T-cell-APC cross-talk in our murine model of metal-DTH, we blocked costimulatory receptor CD86 in vivo. Metal-sensitized C57BL/6 mice were administrated anti-CD86 antibody in vivo during the course of metal-sensitization. Anti-CD86 treated metal-sensitized mice showed a significant decrease in paw inflammation upon Ni challenge (p<0.0001; **Fig 7A**). Additionally, splenic CD4^+^ T-cells isolated from anti-CD86 treated metal-sensitized mice displayed significantly reduced Ni- and Co-specific T cell proliferation responses (p<0.0001) compared to metal-sensitized mice that did not receive any treatment (**Fig 7C**). This data indicates that in this murine model of metal-DTH, co-stimulatory/secondary signals through CD86 by APCs are necessary for sufficient antigen signal strength during priming to elicit effector metal-reactive CD4+ T cells.

### Effect of IL-17A blockade in vivo on metal-DTH responses

**Fig 8:**
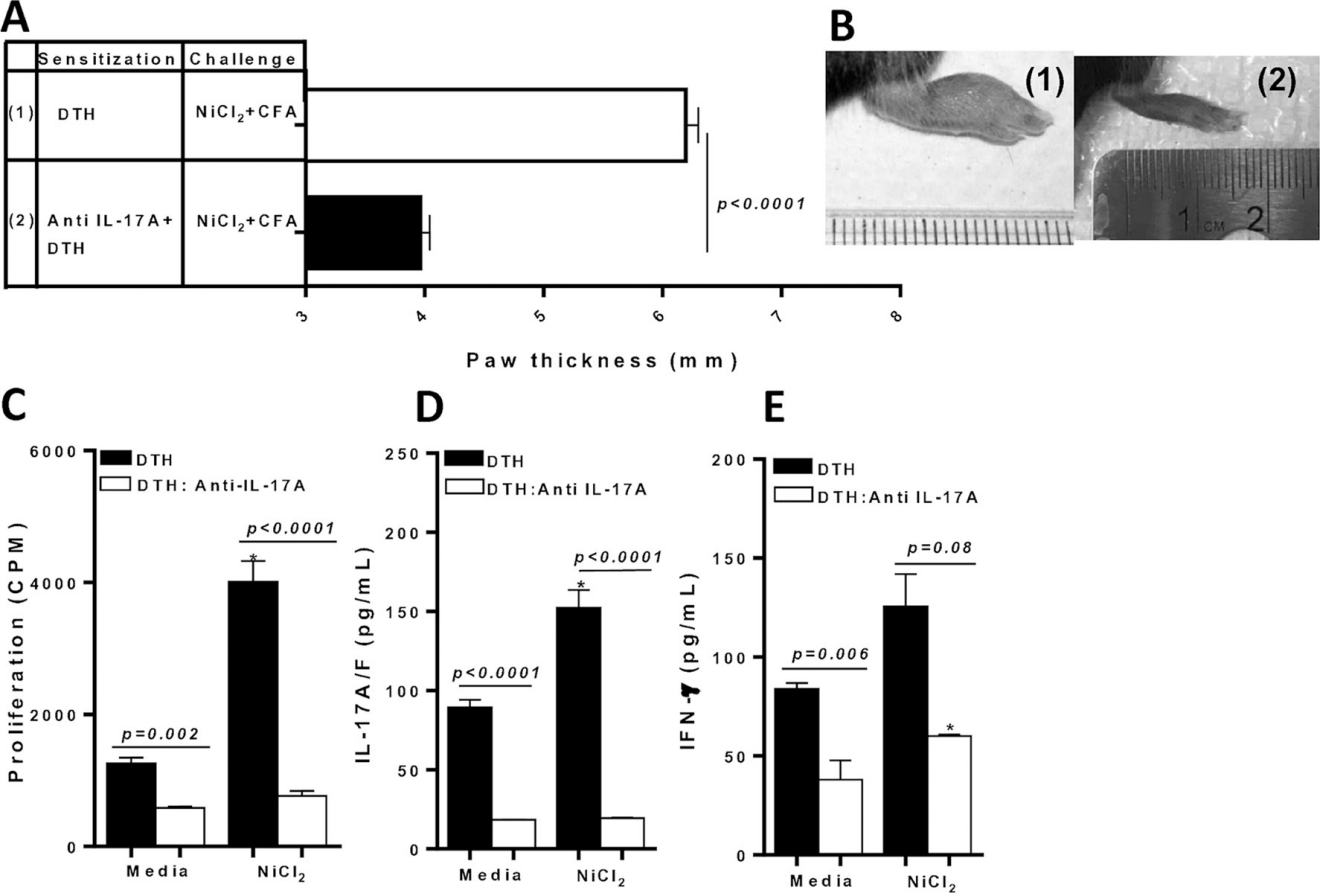
Inhibiting IL-17A bioactivity in vivo in metal-sensitized C57BL/6 mice effectively mitigates metal reactivity. (A) DTH was determined by measuring paw thickness 48 h after challenge in C57BL/6 mice that were sensitized and challenged as indicated on D 12. Sensitized mice in group 2 received systemic ip injection of Anti-IL-17A prior to metal sensitization and every 48 hrs until D 14. (B) Corresponding representative photographs of inflammatory lesions in the paw of metal-DTH treated mice on D 14. At D 14, spleens were harvested. (C) CD4+ T cells were purified from the spleens of either DTH C57BL/6 (black bars) or DTH Anti IL-17A treated C57BL/6 mice (white bars) and co-cultured with mitomycin-C treated naïve total spleen cells from C57BL/6 with Ni (0.001mM) challenge for four days. Proliferation of CD4+ T cells was measured by ^3^H-thymidine incorporation. Supernatants were harvested and assayed by ELISA for (D) IL-17A/F and (E) IFN-γ. Data represent one of three independent experiments with 3–4 mice/group in each experiment. Data are shown as mean ± SEM. Statistical significance was determined by Student’s unpaired two-tailed t-test (* P≤0.05).

Since an increase in IL-17A/F production by CD4+ T cells from metal-sensitized C57BL/6 mice correlates with increased metal-DTH responses, we examined if blocking IL-17A in vivo during metal sensitization was effective at mitigating metal-DTH reactivity in vivo and in vitro. We found that metal-sensitized C57BL/6 mice that received systemic anti-IL-17A treatment, had significantly less paw inflammation upon Ni challenge compared with metal-sensitized mice that did not receive any biologic treatment (p<0.0001; **Fig 8A and 8B**). This observed reduction in vivo of metal-DTH severity correlated with significantly decreased CD4+ T cell proliferation responses to Ni challenge in vitro (p<0.0001; **Fig 8C**). Further, CD4+ T cells from anti-IL-17A treated metal-sensitized mice displayed a significant decrease in IL-17A/F production to Ni (p<0.0001; **Fig 8D**). However, IFN-γ secretion was significantly increased to Ni compared to both respective control (**Fig 8E**) and to IL-17A/F secretion to Ni (p<0.0001; Fig 8D vs. 8E). Taken together, these results further demonstrate that metal-DTH responses are mediated by metal-reactive IL-17A/F producing CD4+ T cells. Also, this data shows that IFN-γ production is up-regulated in the absence of IL-17 activity but does not translate/correspond with metal-DTH severity. Alternatively, IFN-γ likely acts as a regulator of IL-17 inflammatory activity, and is likely a mechanism by which adaptive inflammation is controlled in metal-DTH responses.

### *In vitro* metal reactivity of human lymphocytes is mitigated by blocking the NLRP3 inflammasome pathway

**Fig 9:**
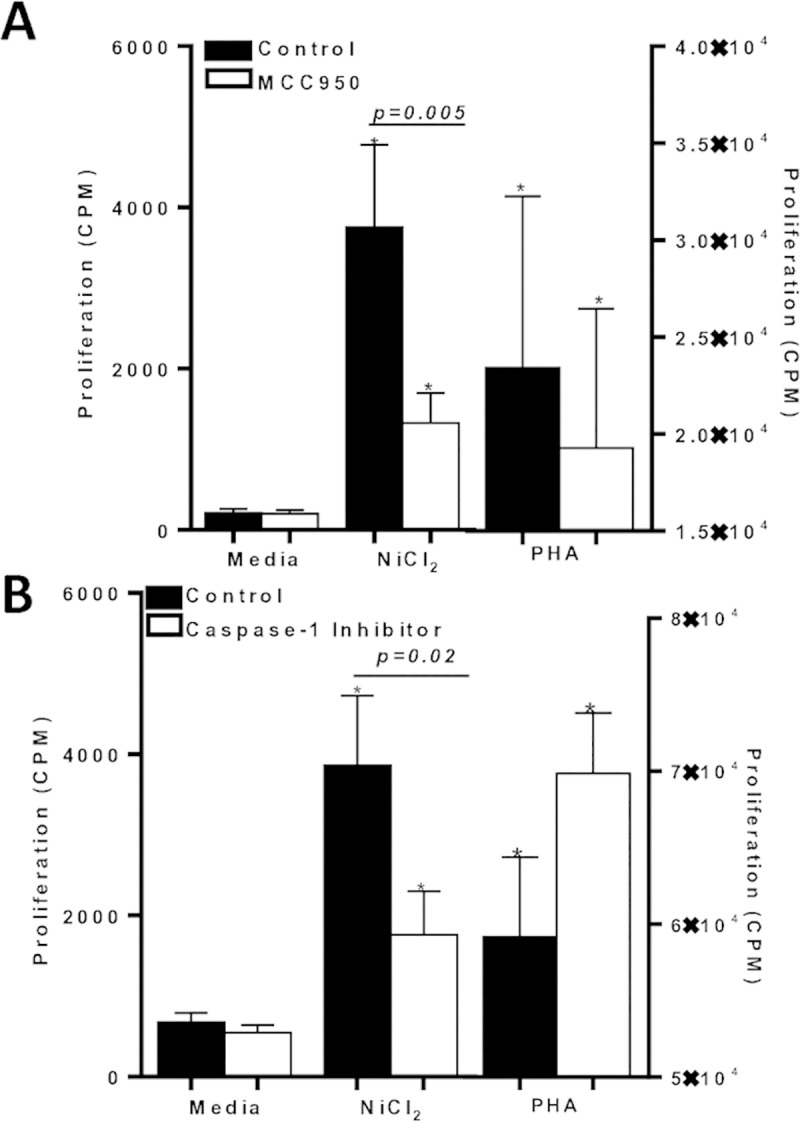
Metal-reactive lymphocytes from individuals’ exhibit significant decreased metal-reactivity in the presence of inflammasome based inhibitors. Proliferation of human lymphocytes was measured by ^3^H-thymidine incorporation after 6 d of in vitro metal-challenge and in the presence of either a: (A) NLRP3 inflammasome inhibitor (MCC950) from n = 5 individuals or (B) Caspase-1 inhibitor from n = 5 individuals. Data are shown as mean ± SEM. Asterisk (*) signifies statistical significance at p≤0.05 compared to respective control group as determined by paired Student’s t-test.

Our findings that metal-sensitized Nlrp3-/- and Caspase-1-/- mice displayed weakened metal-reactive CD4+ T cell activation, which correlated with decreased severity of metal-DTH responses, led us to test possible therapeutic strategies by targeting the inflammasome pathway in metal-reactive individuals in vitro. Peripheral blood mononuclear cells (PBMCs) were isolated from individuals and co-incubated with an array of inflammasome pathway inhibitors in the absence/presence of metal challenge for total of 6 days, i.e. MCC950 (a NLRP3 inflammasome inhibitor), Caspase-1 inhibitor or IL-1Ra (IL-1 receptor antagonist). Subsequently, person-dependent metal-reactivity was determined by lymphocyte proliferation response to metal challenge. Ni challenged lymphocytes, proliferated vigorously, indicating these individuals are metal-reactive (n = 5; p = 0.006; **Fig 9A**). Whereas, Ni challenged lymphocytes from the same individuals incubated with MCC950 (NLRP3 inflammasome inhibitor) showed only a weak proliferative response to Ni (Fig 9A). Further testing using Caspase-1 inhibitor (Caspase-1 is downstream of the inflammasome complex), significantly reduced lymphocyte reactivity to Ni in metal-reactive individuals (n = 5; p = 0.02; **Fig 9B**). Taken together, these results indicate that in part, NLPR3 inflammasome and caspase-1 activity are required for efficient priming of T cells to implant metals in metal reactive individuals.

**Fig 10:**
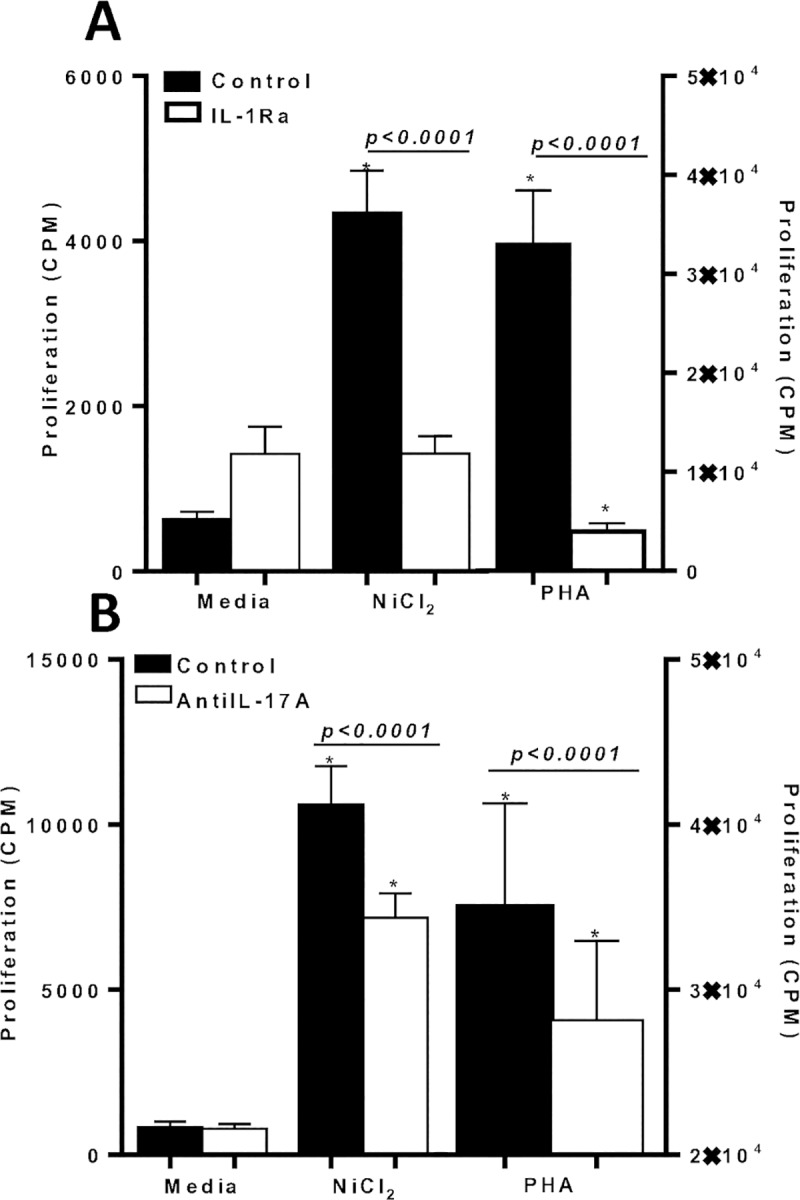
Metal-reactive lymphocytes from individuals’ exhibit significant decreased metal-reactivity when IL-1 and IL-17A bioactivity is inhibited *in vitro*. Proliferation of human lymphocytes was measured by ^3^H-thymidine incorporation after 6 d of in vitro metal-challenge and in the presence of (A) IL-1Ra (IL-1 receptor antagonist) from n = 16 individuals or (B) Anti IL-17A from n = 11 individuals. Data are shown as mean ± SEM. Asterisk (*) signifies statistical significance at p≤0.05 compared to respective control group as determined by paired Student’s t-test.

IL-1 targeted drugs, such as IL-1Ra (Anakinra), are highly efficacious drugs for treating autoinflammatory diseases. To test if IL-1Ra is a possible therapeutic strategy to treat metal-DTH responses in TJA patients, metal-reactive individual lymphocytes were co-incubated in vitro with IL-1Ra in the presence of metal challenge. The addition of IL-1Ra significantly reduced lymphocyte proliferative responses to metal challenge from metal reactive individuals (n = 16; p<0.001; **Fig 10A**). Further, IL-1Ra was the most effective inflammasome based inhibitor (vs. MCC950 or Caspase-1 inhibitor) to mitigate lymphocyte metal reactivity, since, lymphocyte proliferation to Ni with IL-1Ra treatment, was non-significantly different compared to media challenge. Whereas, metal challenged lymphocytes treated with either MCC950 or Caspase-1 inhibitor, displayed significant increases in cell proliferation compared to respective media control.

### *In vitro* metal reactivity of human lymphocytes is mitigated by blocking IL-17A activity

We next asked whether the decrease in metal-DTH responses to anti-IL-17A treatment in vivo using our murine model, would also apply to metal reactive individuals, lymphocytes were co-incubated with anti-IL-17A in the presence of metal challenge (**Fig 10B**). Metal-reactive individuals demonstrated significant decreased lymphocyte proliferation reactivity to Ni in the presence of anti-IL-17A (n = 11; p<0.0001; **Fig 10B**). However, in comparison to the tested inflammasome based inhibitors (Figs 9A, 9B and 10A), anti-IL-17A treatment was not as effective in mitigating lymphocyte proliferation to metal challenge. This could be a consequence of greater immune activation to metal challenge, as exhibited by a robust lymphocyte proliferation from this subset of tested individuals (mean cpm to Ni = 10,600) compared with other individuals tested for metal reactivity (mean cpm to Ni is approximately 4,000 for other groups; Figs 9A, 9B and 10A).

### Metal reactive human lymphocytes exhibit increased IL-17A/F production, but is suppressed by inflammasome based inhibitors

**Fig 11:**
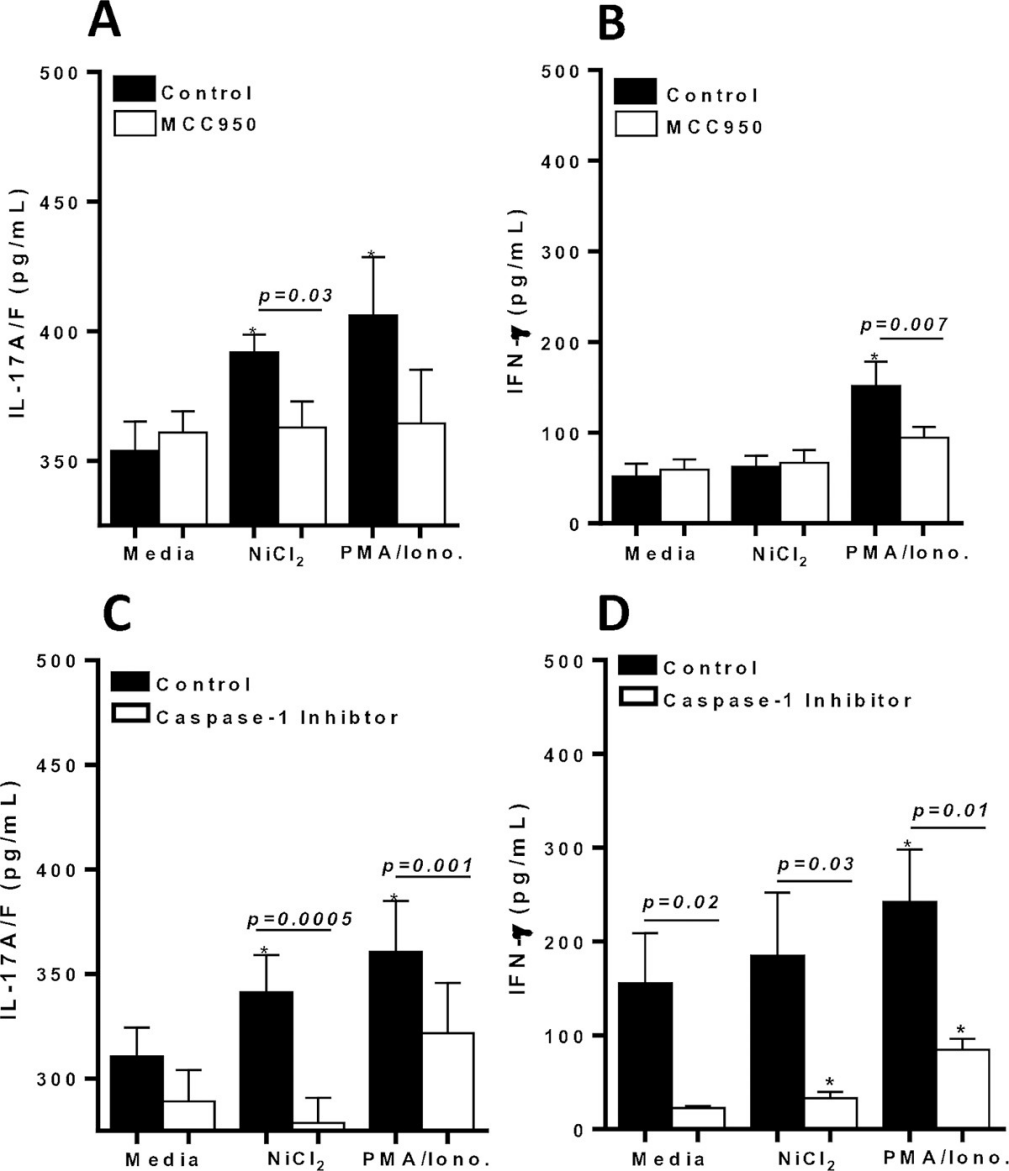
Metal-reactive lymphocytes from individuals’ exhibit significant production of IL-17A/F to metal challenge. Supernatants were harvested on D 6 and assayed by ELISA for production of (A) IL-17A/F or (B) IFN-γ in the absence/presence of metal challenge and/or MCC950 (NLRP3 inhibitor) from n = 3 metal reactive individuals lymphocytes, and (C) IL-17A/F or (D) IFN-γ in the absence/presence of metal challenge and/or Caspase-1 inhibitor from n = 5 metal reactive individuals lymphocytes. Data are shown as mean ± SEM. Asterisk (*) signifies statistical significance at p≤0.05 compared to respective control group as determined by paired Student’s t-test.

To evaluate the role of CD4+ Th17 dependent IL-17A/F and CD4+ Th1 dependent IFN-γ production in metal reactive individuals, cytokine responses were quantified from supernatants of metal challenged lymphocytes in vitro. IL-17A/F secretion was significantly increased from metal-reactive lymphocytes to metal-challenge compared to control values (**Fig 11A**). While IFN-γ production from the same subset of isolated human lymphocytes was non-significantly different to metal challenge compared with control (**Fig 11B**). These results were corroborated by another set of metal-reactive individuals’ lymphocytes that exhibited significant increased IL-17A/F production, but not IFN-γ production to metal challenge in vitro (**Fig 11C and 11D**). However, IL-17 production from lymphocytes to metal challenge, was significantly suppressed by both MCC950 (NLRP3 inflammasome inhibitor) and Caspase-1 inhibitor (Fig 11A and 11C). Lymphocytic production of IFN-γ was not affected by the addition of MCC950 to metal challenge (Fig 11B). Whereas, Caspase-1 inhibitor was a more potent inhibitor that significantly suppressed both IL-17 and IFN-γ production to metal challenge (Fig 11C and 11D).

**Fig 12:**
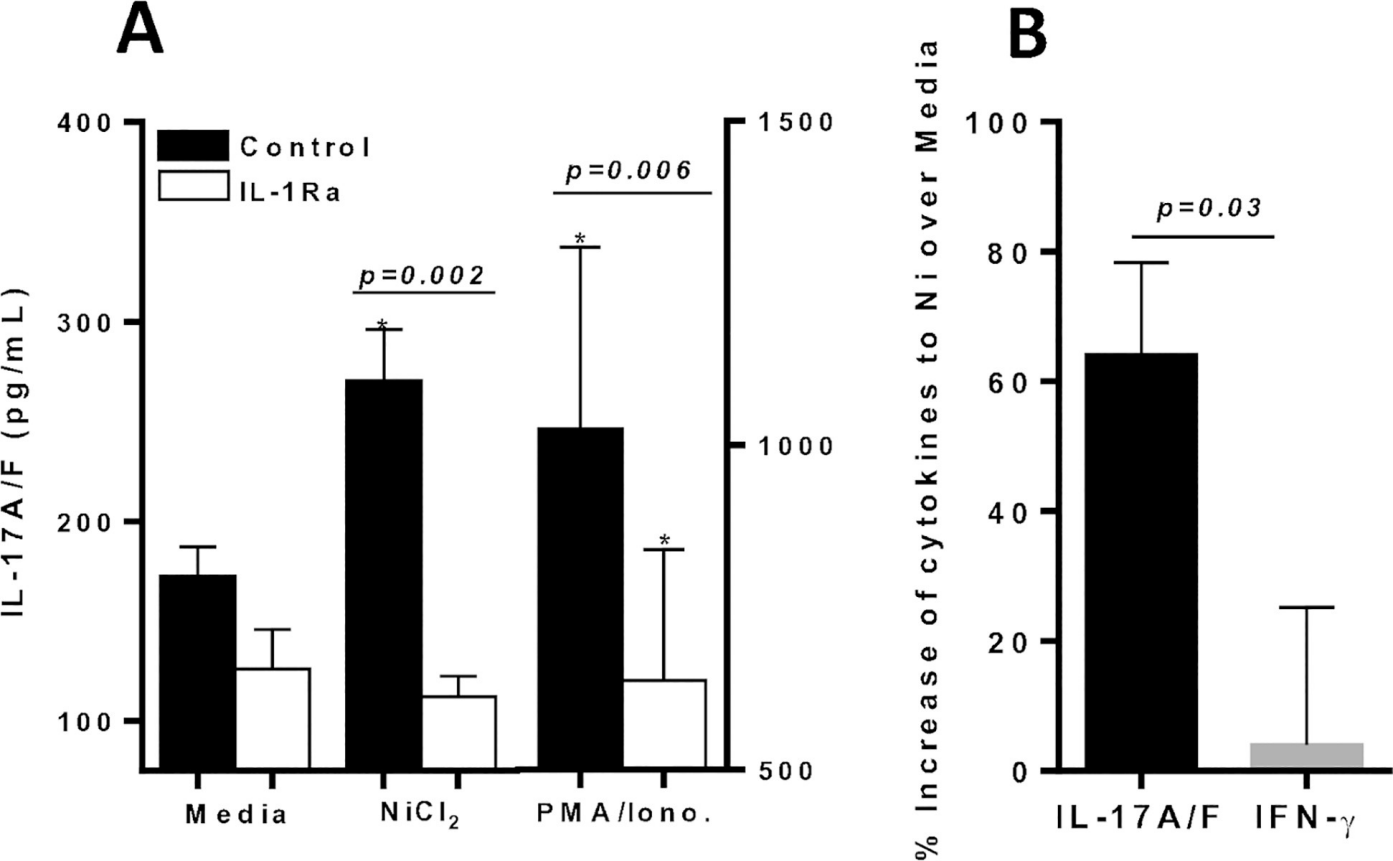
Metal-reactive lymphocytes from individuals’ exhibit significant production of IL-17A/F compared with IFN-γ production to metal challenge. Supernatants were harvested on D 6 and assayed by ELISA for production of (A) IL-17A/F secretion in the absence/presence of metal challenge and/or IL-1Ra from n = 4 metal reactive individuals lymphocytes, and (B) percentage increase of secreted cytokines from n = 4 metal-reactive individuals lymphocytes to Ni challenge over respective control values in the absence of IL-1Ra, demonstrating significant increase in IL-17A/F production compared to IFN-γ. Data are shown as mean ± SEM. Asterisk (*) signifies statistical significance at p≤0.05 compared to respective control group as determined by paired Student’s t-test.

Additionally, metal-reactive individual lymphocytes tested with IL-1Ra in vitro, exhibited significant increases in IL-17A/F production to metal-challenge, but IL-17 production was significantly suppressed by the presence of IL-1Ra (p = 0.002; **Fig 12A**). While IFN-γ lymphocyte responses to metal challenge displayed relatively low detection limits and was minimally affected by the presence of metal in vitro (**Fig 12B**).

Taken together, these results demonstrate that metal reactive individuals’ exhibit IL-17A/F dominant reactivity in metal-DTH responses, which can be significantly suppressed by the use of inflammasome based inhibitors. Moreover, our data demonstrates that the inflammatory environment induced by implant metals affects the function of effector T cells. Specifically, the suppressed production of IL-17 due to the presence of inflammasome inhibitors suggests that inflammatory conditions are essential for the activity of metal-specific effector T cells.

### Inhibition of IL-17A bioactivity suppresses cytokine production in metal-reactive lymphocytes

**Fig 13:**
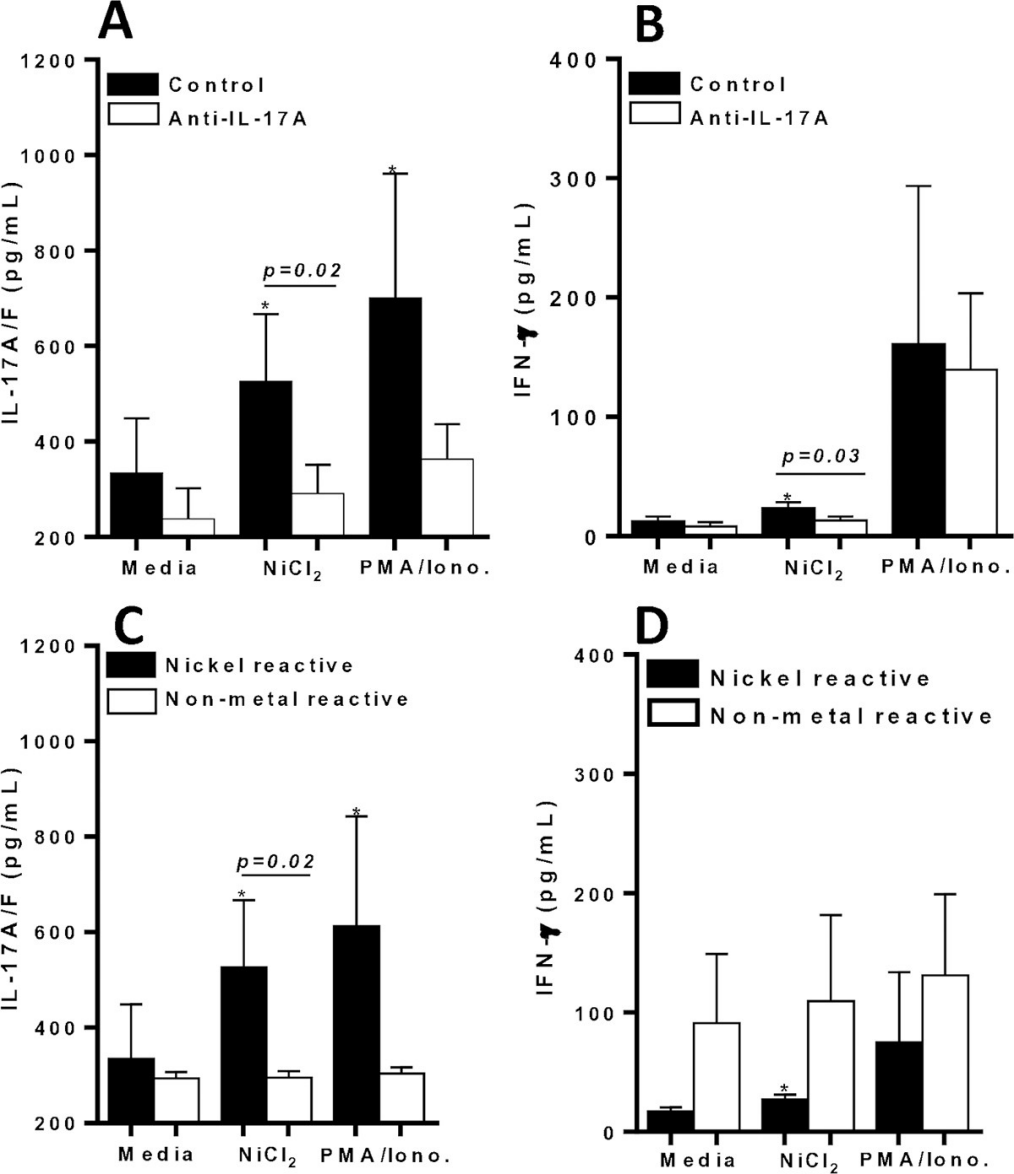
Inhibition of IL-17A signaling mitigates cytokine production in metal-reactive lymphocytes, while non-metal reactive lymphocytes do not exhibit increases in cytokine production compared to metal-reactive individuals in response to metal challenge *in vitro*. Anti-hIL-17A treatment significantly mitigates cytokine production among metal-reactive individuals’ lymphocytes to metal challenge. Production of (A) IL-17A/F or (B) IFN-γ in the absence/presence of metal challenge and/or anti-hIL-17A from n = 10 metal-reactive individuals, and (C) IL-17A/F or (D) IFN-γ production in the absence/presence of metal challenge from n = 10 metal reactive individuals’ lymphocytes vs. n = 9 non-metal reactive individuals’ lymphocytes. Data are shown as mean ± SEM. Asterisk (*) signifies statistical significance at p≤0.05 compared to respective control group as determined by paired or unpaired Student’s t-test.

Since metal-reactive individuals demonstrate increased IL-17 production and inhibiting IL-17 activity reduced lymphocyte proliferation reactivity to metal challenge (Fig 10B), we next assessed the effect of blocking IL-17A in vitro on cytokine production by metal reactive lymphocytes. We found that Ni challenged lymphocytes induced significant production of IL-17A/F (n = 10; p = 0.03; **Fig 13A**), while IFN-γ production was modestly significantly increased compared to respective control values (p = 0.05; **Fig 13B**). Further, IL-17A/F production was significantly greater than IFN-γ responses to Ni by metal-reactive lymphocytes (p = 0.005; Fig 13A vs. 13B). The addition of anti-IL-17A in vitro to metal challenge, significantly reduced both IL-17 and IFN-γ production to metal challenge. This data further confirms that IL-17 bioactivity is centrally involved in the severity of metal-DTH responses. Also, our results show that in addition to inflammasome based inhibitors, inhibition of IL-17A bioactivity is an effective means to mitigate metal-DTH responses among metal reactive individuals.

### IL-17A/F and IFN-γ production in response to metal challenge in non-metal reactive individuals’ lymphocytes

To further identify potential differences in susceptibility to metal-DTH responses in individuals, we assessed the cytokine prolife of non-metal reactive individuals (n = 9). Non-metal reactive individuals were measured by non-significant increases in lymphocyte proliferation to metal challenge compared to respective control values as determined by a LTT assay (data not shown; SI<2, p>0.05). Metal reactive (n = 10) vs. non-metal reactive individual lymphocytes demonstrated inherent differences in cytokine secretion in response to metal challenge (**Fig 13C and 13D**). IL-17 production was significantly decreased in non-metal reactive individuals (p = 0.02; Fig 13C). Whereas, IFN-γ production was non-significantly elevated in non-metal reactive individuals compared with metal reactive individuals (Fig 13D). Thus, these data demonstrate that non-metal reactive individuals do exhibit differences in their cytokine production to metal challenge when compared to metal-reactive individuals. This may account in part, as to why a subset of TJA patients develop metal-DTH responses.

## Discussion

We showed that NLRP3 inflammasome/caspase-1 activity and processed cytokine IL-1β promote adaptive IL-17 production by CD4+ T cells that drive metal-DTH responses to metal that can be released by orthopedic implants. We used an in vivo murine model of metal-DTH in this study as one way to determine the pathomechanisms(s) underlying metal-DTH responses to total joint arthroplasties (TJAs). DTH to implant metals was induced in wild-type C57BL/6, Nlrp3-/- and Caspase-1-/- mice by systemically and locally administrating clinically relevant implant metals [58]. We found that exposure to metal ions in vivo requires processing by an active inflammasome/caspase-1 complex in innate immune cells to induce metal-specific effector T cells in metal-DTH. Inhibition of the inflammasome complex suppressed metal-reactive CD4+ T cells and corresponding IL-17A/F responses to implant metal, which significantly weakened metal-DTH responses both in vivo and in vitro. Furthermore, we found that metal-sensitized Caspase-1-/- mice exhibited increased IFN-γ production, and suppressed IL-17 production. Despite, this increase in IFN-γ expression it did not result in metal-DTH inflammation and lymphocyte proliferation responses. This data supports the general hypothesis that inflammasome activity is required for IL-17 secreting CD4+ Th17 cells. More specifically, the results of this study support our hypothesis that inflammasome activation complex is central to the pathogenesis of Th17 mediated metal-DTH responses to TJAs.

**Fig 14:**
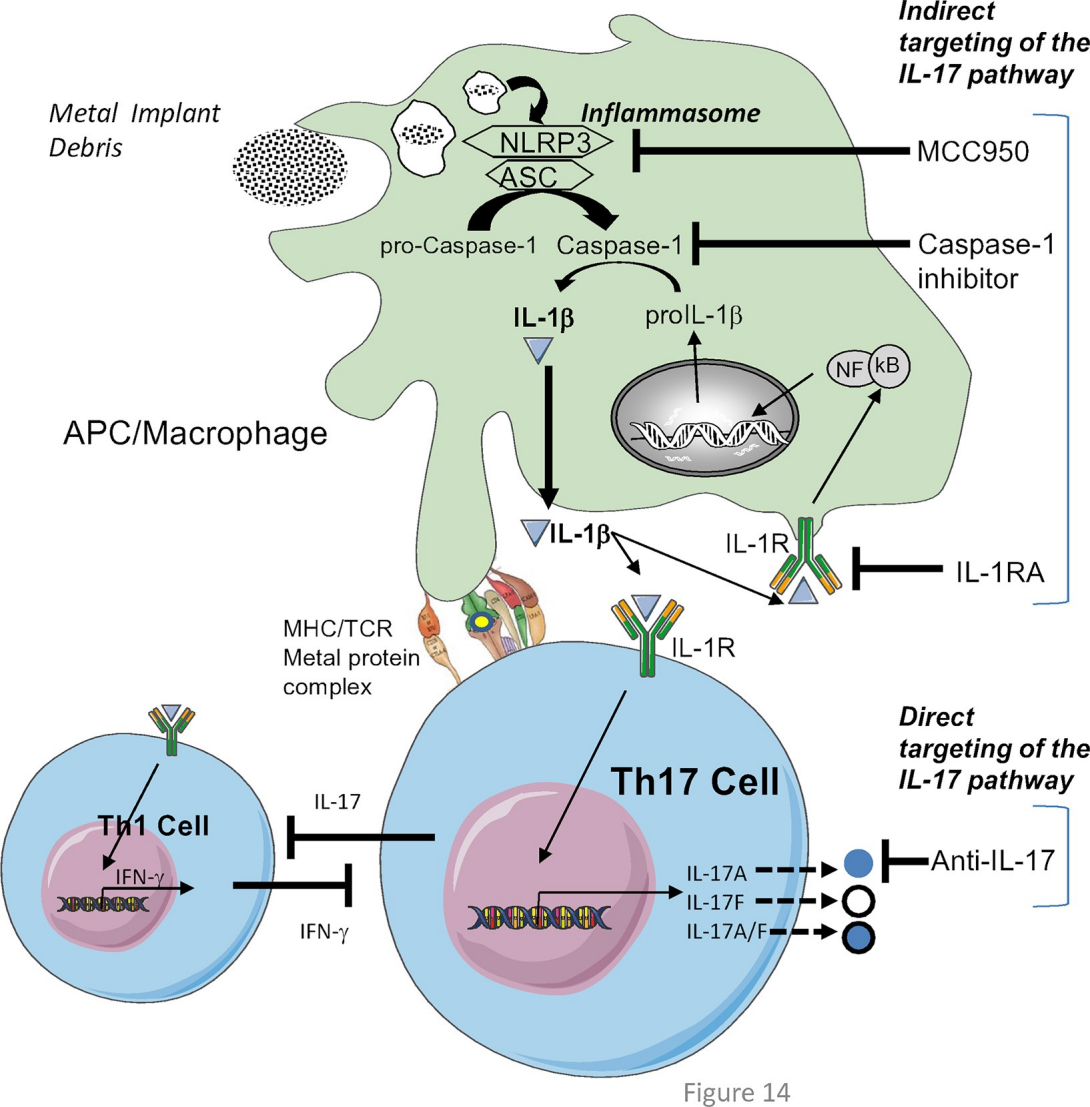
Graphical abstract depicting indirect and/or direct targeting of the IL-17 pathway to mitigate metal-DTH reactivity. A graphical representation of NLRP3 inflammasome activation in APCs by phagocytosis [64] of metal implant debris, which promotes IL-1β signaling and the induction of a Th17-cell phenotype. And the possible biological targets that indirectly (via the inflammasome pathway) and directly (anti-IL-17A) target the IL-17 signaling pathway to decrease the severity of metal-DTH immune reactivity given our findings. IFN-γ is a signature cytokine of Th1 cells and is an important Th1 inducer, that can suppress Th17, Th2 and Treg cell differentiation [47]. While IL-1 and IL-17 are important for Th17 cell differentiation and can repress Th1 cell differentiation. Under metal-DTH responses, IL-1 and IL-17 are highly secreted, indicating more of a Th17 cells phenotype for metal-DTH immune reactivity.

Metal exposure occurs in a variety of settings. The release of particles and metal ions from certain types of metallic orthopedic implants can cause >100 fold elevations in systemic level of metals such as cobalt and chromium [6, 59–61]. It is well established that implant wear-debris stimulates local macrophage recruitment and activation. More importantly, macrophages are the central producers of IL-1β, IL-6 and TNF-α, which are three potent pro-inflammatory cytokines that have been shown to contribute to implant pathologies such as osteolysis and aseptic loosening when stimulated by particulate implant debris [56, 62–64]. We have previously reported that the NLRP3 inflammasome complex is a central mechanism by which macrophages sense and respond to endotoxin-free wear-debris including both particulate and soluble implant debris [56, 62–64]. Further, we have shown that implant debris induces human macrophage production of inflammatory cytokines that leads to up-regulation of surface T-cell costimulatory molecules, thus amplifying a metal-induced adaptive immune response [56]. The degree to which macrophages may be responsible for priming/triggering adaptive immune responses in this context remains unknown. That there is a complex interplay between the two (innate and adaptive) in the context of implant debris DTH reactions to TJAs, has been evidenced histologically with observations of increased peri-implant lymphocyte infiltration/accumulation(s) surrounding peri-implant tissue(s); including CD3^+^ and CD4^+^ T lymphocytes as well as innate immune cells such as CD11c^+^ macrophages [14, 65, 66]. However, there is a lack of therapeutic options for implant associated metal-DTH pathology. Given the increasing number of TJAs performed per year and the decreasing age of TJA patients, a better understanding of the interplay between the innate and adaptive immune system is central to for understanding metal-DTH etiology/pathogenesis and effective therapeutic strategies (**Fig 14**).

Therefore, to determine the pathomechanism(s) underlying metal-DTH responses to TJAs, we used an in vivo murine model of metal-DTH. In vivo experiments using metal-sensitized wild-type C57BL/6 exhibited metal-DTH reactivity that corresponded with robust CD4+ T cell proliferation and increased IL-17A/F responses to metal re-challenge. While metal-sensitized Nlrp3-/- and Caspase-1-/- mice resulted in significant mitigated CD4+ T cell responsiveness to metal re-challenge as measured by proliferation and IL-17A/F production. Surprisingly, metal-sensitized Caspase-1-/- mice revealed enhanced protection from developing metal reactivity compared to NLRP3-/- mice. These results indicate: 1) that the inhibition of the inflammasome pathway is an effective means to mitigate both the sensitization and elicitation phases of metal-DTH responses, and 2) downstream components of the inflammasome (i.e. Caspase-1) demonstrated a greater effect on modulating/inducing T-cell effector mediated immune reactivity to metal allergens (Fig 14).

In assessing cytokine expression by CD4+ T cells from metal-sensitized C57BL/6 mice, we found significant increased expression of IL-17A/F but not of IFN-γ in response to metal challenge in vitro. In contrast, CD4+ T cells from metal-sensitized Caspase-1-/- mice exhibited significant increases in IFN-γ production but not of IL-17A/F production. To further assess the role of IL-17, we used a neutralizing antibody against IL-17A in vivo during the course of metal-DTH induction in C57BL/6 mice. We found that metal-DTH responses are dependent on IL-17 given that neutralization of IL-17 significantly inhibited metal-DTH responses, as measured by in vivo paw inflammation and in vitro T cell proliferation responses (Fig 8). These findings indicate that in addition to inflammasome signaling, IL-17 plays a crucial role in the development of metal-DTH responses to TJAs by activating metal-specific CD4+ T cell responses [67, 68].

Several autoimmune disease models (i.e. experimental autoimmune encephalomyelitis (EAE) and collagen-induced arthritis (CIA)) have demonstrated that IFN-γ has the ability to downregulate symptoms of disease in animal models that use CFA in the induction of the disease [69–72]. Further, it was discovered that in the absence of IFN-γ, there is a loss of control over IL-17 dominated innate and adaptive immune responses [69–74]. Our findings that metal-sensitized Caspase-1-/- mice did not develop metal-DTH responses but exhibited elevated IFN-γ expression, implies that despite the pro-inflammatory nature of IFN-γ, it plays a non-activating or protective role in metal-DTH pathology.

The inflammasome is known to detect multitude of danger signals, such as endogenous molecules released during tissue damage that induce innate immune activation and inflammation. Consequently, the development of DTH immune reactivity to orthopedic metal(s) likely involves a one-two punch where implant debris acts as a danger signal to activate innate immune NLRP3 inflammasome and antigenic to elicit effector T-cells in the sensitization phase.

Consequently, effector T-cells induce inflammation and promote progressive tissue damage at the peri-implant interface during the elicitation phase and upon re-exposure to metal-allergen. This was supported in this study by assessing histological differences from the paw tissue of metal-sensitized C57BL/6 mice. The common, although qualitative feature was the association of lymphocytic infiltration (i.e. aseptic lymphocyte associated vasculitis, ALVAL) corresponding with increased paw erythema and inflammation to metal re-challenge (Fig 1). These results are similar to histological evaluations of failed metal-on-metal total hip replacements that exhibited increased per-implant lymphocyte infiltration/accumulation surrounding the peri-implant tissue ALVAL [9–16]. Additionally, we observed that co-stimulatory molecule CD86 expressed by APCs plays a central role during the initiation of T-cell immune responses to implant metals. In vivo blockade of CD86 during metal-sensitization lead to decreased metal-DTH reactivity that corresponded with weakened T cell proliferative responses to metal challenge (Fig 7). These data strongly indicate that in vivo exposure to implant metals elicits lymphocyte activation and that APC co-stimulation is crucial to the development of metal-DTH lymphocytic immune responses in the murine model of metal-DTH.

The respective roles of Th1, Th2 and Th17 cells in metal-DTH reactions have yielded conflicting data, since each have been indicated to play a role [29–32, 43, 75–77]. Possible explanations for the differences among the studies was the use of patch test vs. LTT assay to determine metal reactivity, and various methods of measuring cytokine expression from blood and skin-derived lymphocytes. Further, studies examining human allergic contact dermatitis reactions to metals is not similar to metal-DTH responses to TJAs, given that the location of the immune response is uniquely different, resulting in differential activation of effector T cell phenotypes which may in part be responsible for observed differences. In this regard we were able to demonstrate that lymphocytes isolated from metal-reactive individuals produced cytokines that correlated with those produced from our model of metal-sensitized C57BL/6 mice and support a Th-17 phenotype dominated response [78]. This data is important in building a consensus of reported DTH pathomechanisms responses which is central to hypothesis validation over time. Importantly, the histological manifestations of metal-DTH reactions were similar, corresponding with lymphocytic infiltrations identified in failed MoM THAs. Metal-reactive lymphocytes treated with inflammasome based inhibitors or anti-IL-17A antibody, exhibited significant decrease in T cell proliferation responses and cytokine production to metal challenge in vitro. These data further support our hypothesis that inflammasome/caspase-1 and IL-17A/F bioactivity is required for the activation of metal-reactive T-cells and the pathogenesis of metal-DTH responses.

An important limitation of the study is that we did not determine if metal sensitive individuals have genetic/epigenetic variations in NLPR3 inflammasome, IL-17 and/or anti-inflammatory (i.e. IL-10, IL-1Ra, IL-4 etc.) gene expression that render them susceptible to metal-DTH immune reactivity vs. non-metal sensitive individuals [79]. However, our in vitro results imply that among metal-reactive lymphocytes that either inhibition of the inflammasome and/or IL-17 pathway is a viable therapeutic strategy to prevent/mitigate implant debris related metal-DTH responses. However, it is important to note that use of inhibitors in vitro can cause potential off-target effects that may in part account for reduced metal-reactivity yet remains potential therapeutic candidates. Additional studies performed with TJR patients that exhibit metal-sensitivity and/or ALVAL, are needed to further establish the relative contribution/role of IL-17 and of Th17 cells in metal-DTH responses and implant outcomes. Future efforts are needed and underway with detailed immunostaining techniques to further characterize the phenotype of the lymphocytic infiltrations (i.e. CD3, CD4, CD8, CD25, CD80/86, CD69, CD19, CD20 etc.) present in the murine paw and expression pattern of co-localized inflammatory cytokines associated with metal-DTH responses. In general, this data may help us better understand disparate clinical responses to TJAs, to better individualize therapy choice.

In summary, our findings suggest that the transition from metal-DTH resistance to susceptibility may be facilitated by active danger signaling, i.e. inflammasome/caspase-1 signaling, and the resulting production of IL-17A/F. This indicates that local release of IL-1β and IL-17A/F near the peri-implant tissue work in concert to promote effector T cell immune reactivity that elicit metal-DTH responses to TJAs. This investigation raises the possibility that excessive NLRP3 inflammasome innate immune reactivity to metal degradation products in vivo can lead to DTH-responses through CD4+ T cell release of IL-17A/F. Targeting the nexus of inflammasome and/or Th17 signaling (e.g. IL-1Ra and/or anti-IL-17) may be a potential treatment option(s) for metal-DTH reactions to TJAs or metal exposure in general.
